# Cocaine-Induced Posterior Reversible Encephalopathy Syndrome (PRES): A Case Report Highlighting Neurological and Clinical Outcomes

**DOI:** 10.15388/Amed.2025.32.1.20

**Published:** 2025-02-18

**Authors:** Patricija Griškaitė, Eleonora Kvaščevičienė, Gabija Laubner Sakalauskienė

**Affiliations:** 1 Vilnius University, Vilnius, Lithuania; 2 Republican Vilnius University Hospital, Vilnius, Lithuania; 3 Centre of Toxicology, Republican Vilnius University Hospital Vilnius University, Faculty of Medicine, Clinic of Anaesthesiology and Intensive Care, Vilnius, Lithuania

**Keywords:** posterior reversible encephalopathy syndrome, cocaine use, stimulant drug use, neurological complications, užpakalinės grįžtamosios encefalopatijos sindromas (PRES), kokaino vartojimas, psichostimuliatorių vartojimas, neurologinės komplikacijos

## Abstract

**Background:**

Posterior reversible encephalopathy syndrome (PRES) is a rare neurological condition characterized by disrupted cerebral autoregulation, often associated with clinical features such as hypertension, encephalopathy, seizures, and visual disturbances. Although it primarily affects females aged 20–65, posterior reversible encephalopathy syndrome can present across diverse demographics. This case underscores the critical importance of identifying uncommon risk factors to facilitate early diagnosis and optimal management.

**Clinical case:**

A 37-year-old male with stage 3 chronic kidney disease secondary to hereditary nephropathy and a history of cocaine and alcohol misuse presented to the emergency department with recurrent seizures, hypertension, hyperthermia and altered consciousness. Imaging demonstrated cortical-subcortical hypodensities on CT and parieto-occipital FLAIR hyperintensities on MRI, consistent with the diagnosis of PRES. A diagnosis of PRES was confirmed based on the patient’s history, neurological evaluation, and characteristic radiological findings.

**Conclusions:**

Raising awareness of PRES and its less recognized but increasingly relevant risk factors, such as stimulant drug use – particularly cocaine – remains a critical aspect of improving diagnostic accuracy and management. Although PRES is typically reversible, delayed diagnosis and treatment may lead to permanent neurological complications, including cerebral infarction and hemorrhage.

## Abstract (it)

**Įvadas:**

Užpakalinės grįžtamosios encefalopatijos sindromas (PRES) yra reta neurologinė būklė, kuriai būdingas smegenų autoreguliacijos sutrikimas, dažnai susijęs su tokiais klinikiniais požymiais, kaip antai hipertenzija, encefalopatija, traukuliai ir regos sutrikimai. Nors dažniausiai serga 20–65 metų moterys, užpakalinės grįžtamosios encefalopatijos sindromas gali pasireikšti įvairioms demografinėms grupėms. Šis klinikinis atvejis pabrėžia retų rizikos veiksnių įvertinimo svarbą, siekiant nustatyti diagnozę ankstyvose stadijose ir užtikrinti optimalų gydymą.

**Klinikinis atvejis:**

37 metų vyras, sergantis trečios stadijos lėtine inkstų liga dėl paveldimos nefropatijos ir piktnaudžiaujantis kokainu bei alkoholiu, atvyko į skubios pagalbos skyrių dėl pasikartojančių traukulių, hipertenzijos, karščiavimo ir sutrikusios sąmonės. Atlikus galvos kompiuterinės tomografijos tyrimą nustatytos žievės ir požievio hipodensinės zonos, o MRT – FLAIR hiperintensinis signalas parieto okcipitalinėje srityje, atitinkantis PRES diagnozę. PRES diagnozė buvo patvirtinta remiantis paciento anamneze, neurologiniu vertinimu ir būdingais radiologiniais duomenimis.

**Išvados:**

Didesnis informuotumas apie PRES ir mažiau atpažįstamus, bet vis svarbesnius rizikos veiksnius, tokius kaip psichostimuliatorių, ypač kokaino, vartojimas, išlieka labai svarbus aspektas siekiant pagerinti diagnostikos tikslumą ir gydymą. Nors PRES paprastai yra grįžtamasis, pavėluota diagnozė ir gydymas gali sukelti ilgalaikių neurologinių komplikacijų, įskaitant smegenų infarktą ir kraujavimą.

## Background

*Posterior Reversible Encephalopathy Syndrome* (PRES) is a rare and often reversible neurological condition caused by disrupted brain blood flow regulation characterized by a combination of both radiological and clinical findings such as hypertension (75–80%), encephalopathy (50–94%), seizures (60–87%), headache (50%), visual disturbances (33–39%), and, less frequently, focal neurological deficits, such as aphasia and hemiparesis (10–19%), and status epilepticus (3–17%) [1,2]. While PRES can affect individuals of any age, it is most commonly observed in young to middle-aged adults, with the majority of cases reported in individuals aged 20–65, predominantly among female patients [3]. The syndrome was first described by Hinchey et al. in 1996 and was initially termed “reversible posterior leukoencephalopathy syndrome” [4]. Over time, PRES has gained significant attention, leading to an increased recognition of cases and a growing understanding of its risk factors. The following clinical case underscores the importance of identifying rare risk factors associated with PRES so that to facilitate timely diagnosis and the initiation of appropriate treatment.

## Materials and Methods

A comprehensive literature review was conducted using the *Medline* (PubMed) database with the keywords ‘posterior reversible encephalopathy syndrome’, ‘PRES’, ‘cocaine’, ‘cocaine use’ and ‘stimulant drugs’, covering a 16-year period from the earliest available publication. A further search was performed on *Google Scholar* by using the same keywords. Only articles in English and with free full text were selected. Following the exclusion of articles that were not relevant to the topic, four case reports specifically focusing on cocaine-induced PRES were identified, alongside 11 selectively chosen articles reviewing posterior reversible encephalopathy syndrome. Furthermore, the anonymized patient’s medical history was reviewed and is presented in the form of a clinical case report.

## Case Report

A 37-year-old male was admitted to Republican Vilnius University Hospital emergency department after being found unresponsive at home. The patient had a history of a prior seizure episode, which was witnessed by a friend. On the way to the hospital, the patient experienced recurrent seizures that were successfully terminated with intravenous administration of 10 mg diazepam. Upon evaluation in the emergency department, the patient was noted to be aggressive, restless, exhibiting altered mental status and experiencing recurrent seizures in the intensive care room. His vital signs included blood pressure of 210/132 mmHg, heart rate of 105 beats per minute, oxygen saturation (SpO_2_) of 97%, and body temperature of 38.4 ºC. The patient was assessed by an attending neurologist, with examination revealing symmetrical and constricted pupils, symmetrical limb movement, and the absence of pathological reflexes. The patient’s status was assessed by using the Glasgow Coma Scale, with a total score of 10 (eye opening: 3, verbal response: 2, motor response: 5). No other abnormalities were noted.

*Anamnesis vitae* includes chronic nephritic syndrome and 3^rd^ stage chronic kidney disease attributed to hereditary nephropathy (podocytopathy, MYF9 gene mutation). Additional comorbidities include hyperuricemia, secondary hyperparathyroidism, and psoriasis. According to the medical records, there was no documentation of the patient currently taking any medications. Two weeks prior to admission, the patient had a known history of alcohol misuse. Furthermore, urine toxicology screening upon admission was positive for cocaine, and it was later disclosed that the patient had been using cocaine intermittently for the previous 10 years.

Laboratory testing revealed neutrophilic leukocytosis, with leukocyte levels at 23.61 × 10^9^/L and neutrophils at 22.48 × 10^9^/L. Renal function markers were moderately elevated, including creatinine and urea. Additional findings included hyperglycemia, marginally elevated aspartate aminotransferase, markedly elevated myoglobin (11.815 μg/L), elevated fibrinogen (9.8 g/L) and prolonged activated partial thromboplastin time. Hypochloremia was also observed.

Over the following days, inflammatory and muscle injury markers showed significant elevation. C-reactive protein (CRP) increased to 299 mg/L from normal levels, procalcitonin (PCT) rose to 3.99 μg/L, and creatine kinase (CK) reached 22.712 U/L.

**Figure 1:**
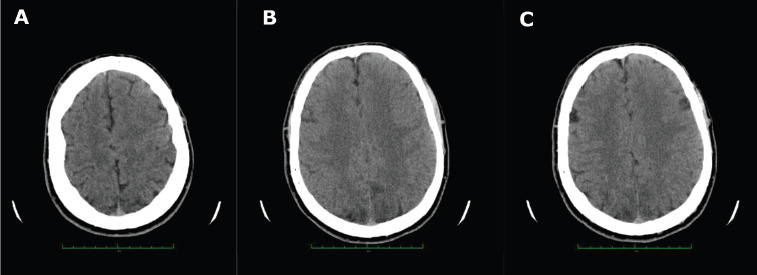
Head computed tomography findings suggestive of PRES: bilateral cortical-subcortical hypodensities in parietal and occipital lobes (A, B, C)

A *Computed Tomography* (CT) head scan demonstrated cortical-subcortical zones of reduced density bilaterally in the parietal and occipital lobes, which are findings suggestive of *Posterior Reversible Encephalopathy Syndrome* (PRES) (Figure 1).

**Figure 2:**
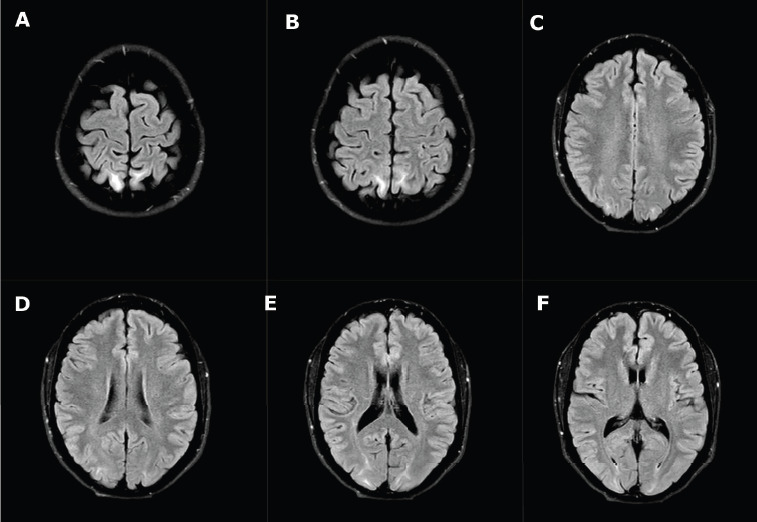
MRI findings revealed FLAIR hyperintense signals in the parieto-occipital subcortical regions, consistent with posterior reversible encephalopathy syndrome (A–F)

A subsequent brain *Magnetic Resonance Imaging* (MRI) revealed *FLuid-Attenuated Inversion Recovery* (FLAIR) hyperintense signal areas in the parieto-occipital subcortical regions, with no reported contrast enhancement or diffusion restriction (Figure 2). These radiological findings are consistent with PRES.

A lumbar puncture revealed normal pressure and clear *CerebroSpinal Fluid* (CSF) without cytosis. However, protein levels were found to be elevated, which is a finding potentially associated with the diagnosis of PRES.

Additional diagnostic workup, including fibrobronchoscopy, chest X-ray, laboratory findings, and sputum culture, confirmed pneumonia caused by *Staphylococcus aureus*.

Based on the patient’s history, including alcohol and drug use, hypertension, chronic kidney disease, and a positive urine toxicology screening for cocaine, along with the neurological evaluation, head CT and brain MRI results, a diagnosis of PRES was confirmed.

The treatment plan included enalapril for hypertension control, sedation with a bolus of propofol in the evening to manage intoxication psychosis, and intravenous as well as infusion administration of diazepam. Antibiotic therapy consisted of vancomycin and tazocin intravenously for acute aspirational pneumonia. Oxygen therapy was initiated via nasal cannulas at 8 L/min to address reduced oxygen saturation (SpO_2_: 92%). Prophylaxis for deep vein thrombosis was provided with low molecular weight heparin. Toward the end of the treatment, the patient was transitioned to oral therapy with nebivolol in the morning and enalapril in the evening.

On the fifth day of hospitalization, during the neurologist’s consultation, the patient’s condition showed notable improvement. No seizures were observed in the intensive care unit. The patient appeared slightly drowsy and reported generalized weakness. The *Glasgow Coma Scale* (GCS) score was 14, the speech was clear, eye movements were unrestricted, the face was symmetrical, the upper limb strength was rated 5/5, and the patient was able to raise and support the legs. No pathological reflexes were observed. Coordination tests were performed adequately.

After nine days of treatment in hospital, the patient was conscious, fully oriented, adequate, with an improved general condition, and with no new complaints. The somatic status was stable and satisfactory. The patient was afebrile, with stable hemodynamics, however, a tendency for hypertension (160/80 mmHg) persisted. Blood tests demonstrated favorable progress. The heart rate was 68 beats per minute with a regular and rhythmic cardiac function. The patient was discharged for outpatient treatment under the supervision and follow-up of a family physician. The patient’s treatment plan upon discharge included antibiotics for acute aspirational pneumonia, alongside antihypertensive therapy combining beta-blockers and *Angiotensin-Converting Enzyme* (ACE) inhibitors.

## Discussion

The association between PRES and cocaine use can be attributed to cocaine’s potent sympathomimetic effects, which provoke vasoconstriction and increase the cardiac output, ultimately resulting in severe hypertension [5]. Cocaine exerts these effects by inhibiting the reuptake of monoamines, including dopamine, serotonin, and norepinephrine, thereby inducing vasoconstriction [5]. Furthermore, the adrenal medulla releases norepinephrine and epinephrine in response to cocaine exposure [6]. Chronic use of cocaine leads to cumulative effects and persistent vasospasm, which amplify the risk of vasoconstriction with each subsequent use and cause sustained endothelial damage [5]. Therefore, cocaine exhibits strong sympathomimetic properties, resulting in substantial vasoconstriction in both central and peripheral circulation [6].

**Graph 1:**
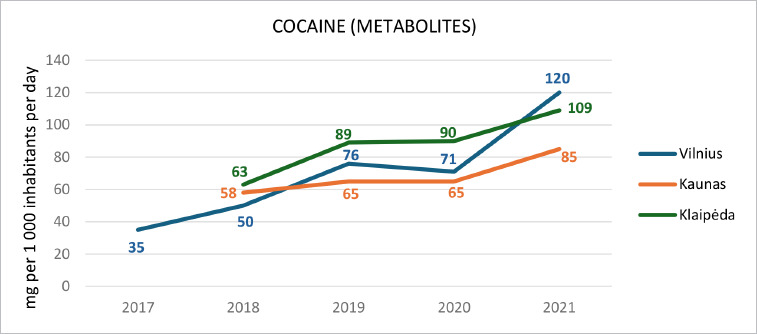
Wastewater analysis results of cocaine metabolites in major cities of Lithuania (2017–2021) [6]

As the prevalence of stimulant drug use increases, particularly cocaine, a notable yet often underrecognized risk factor, the relevance of PRES is becoming increasingly significant. According to 2021 statistics, 14.1% of individuals aged 15–64 in Lithuania reported having used any psychoactive substance at least once in their lifetime, with a prevalence of 1.8% for cocaine use within the same age group [7]. Notably, the overall use of psychoactive substances, including stimulants, has shown a significant increase over the years [7]. For comparison, in 2016, lifetime use of any psychoactive substance in this demographic was 11.6%, with cocaine use reported at 0.7% [7]. Wastewater analysis conducted to monitor the prevalence of illegal stimulant use revealed a gradual increase in the consumption of cocaine over the period of 2017–2021 (Graph 1) [7].

Across Europe, the European Drug Report 2024 highlights a consistent rise in the availability of cocaine over recent years [8]. Cocaine is currently the second most commonly used illicit substance in Europe, following cannabis [8]. In addition, municipal wastewater analysis in two-thirds of cities with available data for 2022 and 2023 revealed a significant increase in cocaine residues, further reflecting the rising prevalence of its use [8]. Surveys conducted across the European Union indicate that nearly 2.5 million individuals aged 15 to 34 years (2.5% of this age group) reported using cocaine in the past year [8]. Among 13 European countries that have conducted comparable surveys since 2021, five reported higher prevalence estimates than in previous studies [8]. These findings, along with other indicators, suggest that the increasing availability of cocaine has expanded both its geographical reach and its social distribution across Europe [8].

Although the pathophysiology of PRES remains idiopathic, several mechanisms have been proposed [8]. The hypertension/hyperperfusion theory suggests that a sudden rise in blood pressure surpasses cerebral autoregulation, leading to arteriolar dilation, hyperperfusion, *Blood-Brain Barrier* (BBB) disruption, and vasogenic edema [9]. The autoregulation hypothesis posits that severe hypertension impairs the cerebral blood flow control, causing vasoconstriction, hypoperfusion, ischemia, and vascular endothelial growth factor-mediated vascular permeability, resulting in edema [9]. Regarding the latter two hypotheses, the previously mentioned sympathomimetic and vasoconstrictive effects of cocaine are of considerable significance. Consistent with these pathophysiological processes, cocaine-induced vasoconstriction and increased cardiac output lead to hypertension, which triggers the hypothesized pathways, with hypertension acting as a key component in the sequence and ultimately resulting in posterior reversible encephalopathy syndrome. The third, endothelial dysfunction, hypothesis proposes that various pathological conditions trigger endothelial dysfunction, activating the immune system and inflammatory cytokines, which disrupt BBB integrity, increase permeability and fluid accumulation within the cerebrovascular endothelium, ultimately resulting in edema [9]. This hypothesis is particularly relevant in explaining the 15–20% of PRES cases that occur in the absence of significantly elevated or only mildly elevated blood pressure [9].

Elevated levels of the markers of endothelial dysfunction, including endothelin-1, tissue plasminogen activator, fibronectin, and particularly von Willebrand factor, have been observed in patients with PRES [3]. These findings underscore the significant role of endothelial injury in the pathophysiology of the condition [3]. Although the above-mentioned blood markers were not evaluated in this case, alternative laboratory findings were consistent with PRES-related symptoms. The patient had significantly elevated levels of myoglobin and creatine kinase, suggestive of rhabdomyolysis, likely resulting from the seizures, which are one of the characteristic manifestations of PRES.

Although PRES can involve any region of the brain, it predominantly affects the posterior cerebral circulation [5]. This predilection is likely due to the relatively reduced sympathetic innervation in this area [5].

The diagnosis of PRES requires a combination of clinical and radiological findings [3,10]. Therefore, it is crucial to perform and compare various diagnostic imaging modalities to identify the characteristic features of PRES [11]. CT scans, often the first-line test in patients with seizures or altered mental status, can reveal bilateral white matter edema in the parieto-occipital regions but have limited sensitivity [9]. If CT findings are inconclusive, MRI should be performed considering its greater sensitivity and specificity, notably due to its superior resolution of posterior fossa structures [1,5,9]. Characteristic MRI findings typically appear as hyperintense signals in the affected regions on T2-weighted imaging, with fluid-attenuated inversion recovery (FLAIR) sequences providing enhanced visualization of septal, peripheral, and cortical lesions [3]. Typical bilateral cortical–subcortical vasogenic edema observed in PRES can be classified into three predominant patterns: parieto-occipital (22%), holohemispheric watershed (23%), and superior frontal sulcus (27%) [1]. *Diffusion-Weighted Imaging* (DWI) aids in differentiating PRES from other conditions such as basilar strokes [1]. In the presented case, post-contrast MRI enhancement was not reported. However, contrast enhancement is observed in 38–50% of PRES cases, and it follows the leptomeningeal, cortical pattern in regions with altered FLAIR signals, or a combined pattern [1]. Additional findings could include intracranial hemorrhages (10–25%) [1]. PRES can also present with a central-variant pattern, characterized by alterations in the brainstem, basal ganglia, posterior limb of the internal capsule, cerebellum, and periventricular regions, without involvement of cortical or subcortical areas [1]. Frontal and temporal lobe involvement is observed in 75% of cases [1].

Additional advanced neuroimaging techniques, such as *Computed Tomography Angiography* (CTA), *Magnetic Resonance Angiography* (MRA), and conventional *Digital Subtraction Angiography* (DSA), are valuable tools for identifying vascular abnormalities in patients with posterior reversible encephalopathy syndrome [9]. These modalities can reveal findings such as vasospasm, arteritis, diffuse or focal vasoconstriction, vasodilation, and the characteristic ‘string-of-beads’ appearance [9].

The primary goals in treating PRES include the control of blood pressure, commonly using diuretics and calcium channel blockers, eliminating the triggering factor, managing symptoms, providing ventilatory support, and administering anti-epileptic drugs when indicated [12]. A gradual reduction in the blood pressure is strongly recommended to avoid exacerbating the condition [12].

PRES is reversible in 70–90% of patients when promptly diagnosed and treated [3]. However, despite the generally favorable outcomes, PRES-related mortality can reach up to 19%, and approximately 44% of patients experience varying degrees of functional disability [13]. Residual neurological deficits, such as epileptiform disorders and motor impairments, are among the noted complications [3,14]. Severe complications, while infrequent, occur in fewer than 10% of cases and include intraparenchymal hemorrhage with mass effect, cerebral herniation secondary to edema, and subarachnoid hemorrhage resulting from endothelial dysfunction [3]. Recurrent episodes of PRES have been observed in fewer than 5% of cases, particularly in individuals with nephrotic syndrome, HIV infection, or those undergoing chemotherapy [3].

Few case reports published in the last decade have investigated the relationship between cocaine use and PRES. Consistent with our findings, these case reports describe recent cocaine use, confirmed through medical history or urine toxicology screening, followed by severe hypertension, clinical features of PRES, and MRI findings demonstrating T2-weighted and FLAIR signal abnormalities consistent with PRES, with or without diffusion restriction [3,5,12,15]. PRES has also been reported in cases associated with other illicit drug use, including mephedrone (a cocaine analog known as ‘the bubble’), amphetamine, methamphetamine, lysergic acid amide and kratom combined with dextroamphetamine [2,16–18]. Our case contributes to the growing recognition of PRES in the context of illicit drug use, especially cocaine, as reported in recent years.

## Limitations

As this report is based on a single patient, the ability to apply these findings to a wider clinical context is limited, highlighting the need for further research with larger cohorts. In addition, the absence of follow-up data limits the evaluation of long-term outcomes, including the possibility of functional impairment, recurrent episodes, and the durability of the treatment effects.

## Conclusions

Although PRES is typically reversible, as suggested by its name, delays in diagnosis and treatment can lead to permanent neurological complications, including cerebral infarction and hemorrhage [12]. Identifying and addressing rare risk factors for PRES is crucial to enable timely diagnosis and the initiation of appropriate treatment. From a radiological perspective, while CT is often the first imaging modality employed in emergency settings, MRI is the preferred method due to its superior sensitivity in detecting PRES [9]. Additionally, raising awareness of PRES and its less-recognized but increasingly relevant risk factors, such as stimulant drug use, particularly cocaine, remains a critical aspect of improving the diagnostic accuracy and management.
